# Construct validity of a figure rating scale for Brazilian adolescents

**DOI:** 10.1186/1475-2891-11-24

**Published:** 2012-04-13

**Authors:** Fernando Adami, Deivis Elton Schlickmann Frainer, Fernando de Souza Almeida, Luiz Carlos de Abreu, Vitor E Valenti, Marcelo Marcos Piva Demarzo, Carlos Bandeira de Mello Monteiro, Fernando R de Oliveira

**Affiliations:** 1 Departamento de Saúde da Coletividade, Faculdade de Medicina do ABC, Av. Príncipe de Gales, 821, 09060650, Santo André, SP, Brazil; 2 Instituto de Saúde Coletiva, Universidade Federal da Bahia, Salvador-Bahia, Brazil; 3 Laboratório de Escrita Científica, Departamento de Morfologia e Fisiologia, Faculdade de Medicina do ABC, Av. Príncipe de Gales, 821, 09060650, Santo André, SP, Brazil; 4 Departamento de Fonoaudiologia, Faculdade de Filosofia e Ciências, Universidade Estadual Paulista, UNESP, Av. Higyno Muzzi Filho, 737. 17, 525900 Marília, SP, Brazil; 5 Universidade Federal de Lavras, Câmpus Universitário, 37200-000 Lavras, MG, Brazil

**Keywords:** Body contour, Adolescents, Validity, Body image

## Abstract

**Background:**

Figure rating scales were developed as a tool to determine body dissatisfaction in women, men, and children. However, it lacks in the literature the validation of the scale for body silhouettes previously adapted. We aimed to obtain evidence for construct validity of a figure rating scale for Brazilian adolescents.

**Methods:**

The study was carried out with adolescent students attending three public schools in an urban region of the municipality of Florianopolis in the State of Santa Catarina (SC). The sample comprised 232 10-19-year-old students, 106 of whom are boys and 126 girls, from the 5th "series" (i.e. year) of Primary School to the 3rd year of Secondary School. Data-gathering involved the application of an instrument containing 8 body figure drawings representing a range of children's and adolescents' body shapes, ranging from very slim (contour 1) to obese (contour 8). Weights and heights were also collected, and body mass index (BMI) was calculated later. BMI was analyzed as a continuous variable, using z-scores, and as a dichotomous categorical variable, representing a diagnosis of nutritional status (normal and overweight including obesity).

**Results:**

Results showed that both males and females with larger BMI z-scores chose larger body contours. Girls with higher BMI z-scores also show higher values of body image dissatisfaction.

**Conclusion:**

We provided the first evidence of validity for a figure rating scale for Brazilian adolescents.

## Background

Body dissatisfaction is a component of body image related to attitudes and evaluations of one's own body [1,2], and is defined as a negative evaluation of one's own body. It is diagnosed by graphical instruments showing figure drawings of the body and parts of it (such as the belly and hips), and through questionnaires containing questions on body weight and other aspects related to body acceptance [3].

Body dissatisfaction is considered as more than an etiologic factor, it is also a risk factor. Moreover, it is also associated with eating disorders such as anorexia, bulimia and binge eating, above all in women [4-7]. Other outcomes related to body dissatisfaction are attempted suicide [8] and depression [9].

During puberty several factors may change the way an individual views his or her own body; changes in body shape during this period play an important role in the issue of body acceptance [10,11]. Relatives, peers, partners and the media also influence body-related issues [12,13].

In girls, prospective studies have shown that perceived pressure to be slim and increased body mass are predictors of body dissatisfaction [3,14]. Recent studies have shown variables such as social comparison and social support (family and friends) to be strong predictors of body dissatisfaction in girls [10,15]. Few studies have been carried out in boys to determine predictors of body dissatisfaction, but one may point out the variables of internalization of the ideal of muscle hypertrophy (Jones) and family support [10].

Considering that figure rating scales were used in previous studies [16,17], the objective of the present study springs from concern with the need for valid methods to measure body image and body dissatisfaction in Brazilian adolescents. Childress and coworkers [18] adapted a scale to evaluate body contour based upon the body contour figures developed by Stunkard & Sorensen [19]. They made that in order to allow the scale to be used in American children and adolescents, although it was not presented with validity data.

The Stunkard & Sorensen scale [19] was validated in Brazil for the adult population [2]. However, no data have yet been published on the validation of the scale for body silhouettes adapted by Childress et al [18]. The objective of this study is therefore to describe evidence of construct validity for the Childress et al. [18] figure rating scale in Brazilian adolescents. The construct validity hypothesis was that heavier adolescents would choose larger body contour shapes; and that in girls, body image dissatisfaction would increase as BMI z-scores rose.

## Method

### Study population

The sample was calculated following these parameters: probability of Type I Error ([[]]) equal to 0.05; probability of Type II Error (β) equal to 0.20 and minimum value of correlation between measurement methods (ρ) equal to 0.3, with an equation for n presented by Machin et al. [20]. Based on these values, 84 adolescents of each gender are needed, and when 20% losses are added, this comes to a total of 101. The research project was approved by the Santa Catarina State University's Committee of Ethics in Research, accredited by the Ministry of Health's Committee of Ethics in Research (CONEP) since 1998. Data-gathering took approximately 60 days, in September and October 2005. Free and informed consent was obtained from and signed by the parents for every child's participation.

The sample comprised 232 10-19-year-old students, from the fifth year of Primary School to the third year of Secondary School, 106 of whom were boys, and 126 were girls, with the following mean values (sd): for boys, 14.4 (2.6) years of age and for girls 13.9 (2.5) years of age. The study was carried out with all students from the above-mentioned range of school years studying in 3 public schools in a neighborhood of Florianopolis, Santa Catarina.

### Construct validation hypothesis

The concept of validity is about how far a given instrument really measures the characteristic it proposes to measure [21].

A construct is a latent abstract variable that is constructed by the researcher in his or her imagination, and is a non-observable dimension [21]. The construct validation process must be thought of within a theoretical context that refers to how much a given measurement relates to others. This relationship derives from hypotheses that are consistent with the concept or construct that is being measured [22].

In the present study, the validity of the construct is given by the hypothesis that the larger the figure drawing identified by the individual, the larger will be the BMI z-score. To test whether there is construct validity, in the present study we correlated data for the contour identified by the child with the BMI z-score. Since two constructs may be present in the same domain, the scores of both variables are expected to present a positive correlation. Furthermore, girls are expected to have greater body dissatisfaction the greater their BMI z-score.

According to Carmines and Zeller [22], construct validity involves three different steps: 1) the relationship between the concepts must be specified (e.g. the higher the number of the contour identified, the greater the BMI z-score; 2) the empirical relationship between the measures of the concepts must be examined (correlation between the contour identified and the BMI z-score); and 3) the empirical evidence must be interpreted in terms of how much it elucidates about the validity of the construct of the measurement of interest (does the correlation between the variables have the expected positive direction?)

### Data-gathering--instruments and measurements

#### Body contour drawings

**Figure 1:**
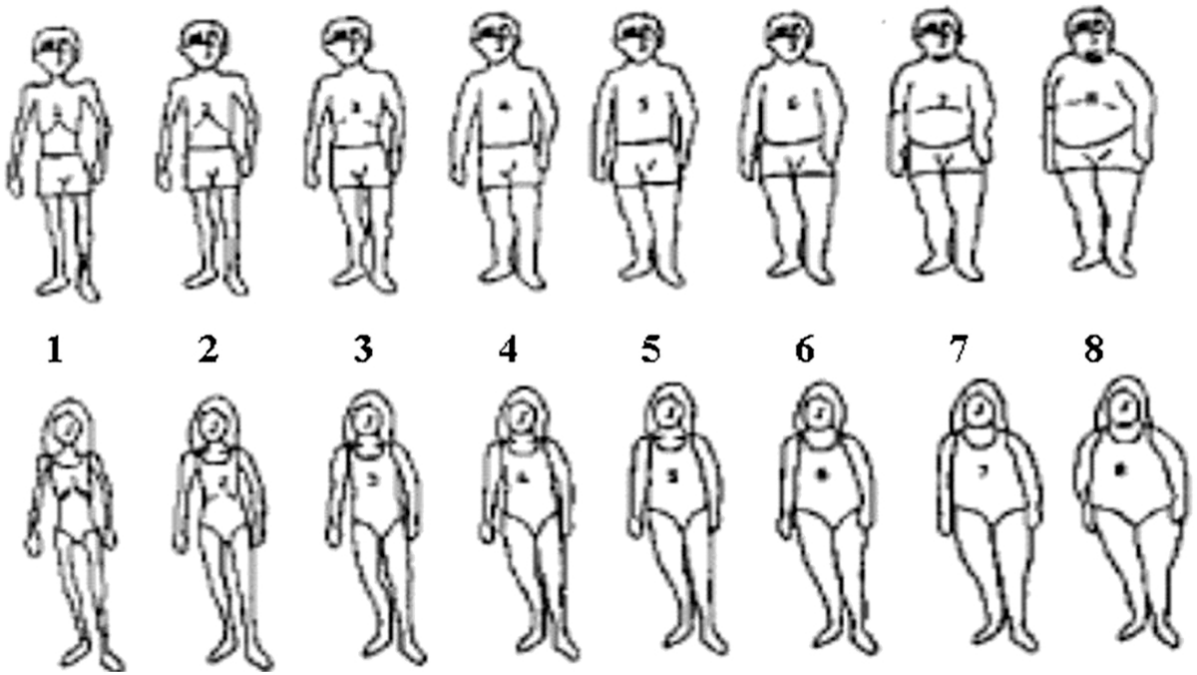
**Figure rating scale proposed by Childress et al**. [18].

The figure rating scale adapted by Childress and coworkers [18] consists of 8 figures representing several children's and adolescents' body outlines, ranging from very slim (contour 1) to obese (contour 8) (Figure 1).

From the eight outline figures available, the schoolchildren chose the one that matched their current body size (CBS - the figure showing the contour they believe they have) and the one matching their ideal body size (IBS - the figure showing the contour the child would like to have); this was done in a private room, the children having had prior individualized explanation given by the chief researcher. Degree of Dissatisfaction with their Body Size (DDBS) was obtained by subtracting the Ideal Body Size (IBS) from the Current Body Size (CBS). The DDBS score showed the degree of dissatisfaction with body shape; the magnitude may be positive when the individual wishes to increase their body size, and negative if they wish to reduce their size. To characterize body dissatisfaction, regardless of whether an individual wishes to increase or reduce their contour, DDBS was also used in a module DDBS, thus: a) dissatisfied - individuals with DDBS > 0 and b) satisfied - individuals with DDBS = 0.

#### Anthropometric Measurements

Weight and height were measured by researchers using internationally accepted techniques [23,24] under supervision of pediatricians from the daycare centers and all data were collected from records of child care using a standardized form. A portable Filizzola electronic scale accurate to within 0.1 kg was used to evaluate body weight. A portable stadiometer accurate to within 0.1 mm was used to evaluate height. The students were weighed and measured barefoot and in light clothing. The measurements were taken by an experienced anthropometrist.

Weights and heights provided the body mass index (BMI), which is weight in kilos divided by the square of the height in meters.

Since BMI varies in childhood and adolescence according to age and gender, the standardized score (z-score) for the BMI variable had to be calculated. The new references for evaluation of the nutritional status of Brazilian children based on the distribution of BMI values [25] were used. To calculate the z-score, LMS values were used, by age and gender, according to the following formula:

$$Z\;\mathrm{score}\;\mathrm{BMI}=\left[{\left(\mathrm{BMI}/M\right)}^{L}-1\right]/\left(LS\right)$$

The LMS sums up the data in smoothed curves that are specific to each stratum, which in this case are the ages and genders. Parameter M is the median value of the index observed inside each stratum; parameter S is the coefficient of variation for each stratum; and parameter L is the Box-Cox coefficient employed for the mathematical transformation of the values of the variable in question in order to obtain a normal distribution in each stratum [26].

Nutritional status was determined according to the criteria of Conde and Monteiro [25] for BMI values by gender and age. The children were thus diagnosed as normal or overweight including obesity.

### Statistical analysis

The data were analyzed by the distribution of frequency of BMI z-scores according to current body shape for boys and girls. Given the non-normality of the data, Spearman's correlation coefficient was used to verify the correlation between BMI z-score and Current Body Size (CBS) and module of degree of body dissatisfaction - DDBS, in both sexes. SPSS 15.0 was the statistical package used.

## Results

No BMI value was greater or lesser than 4 standard deviations, which is an indicator of the biological plausibility and consequent quality of the evaluators.

**Figure 2:**
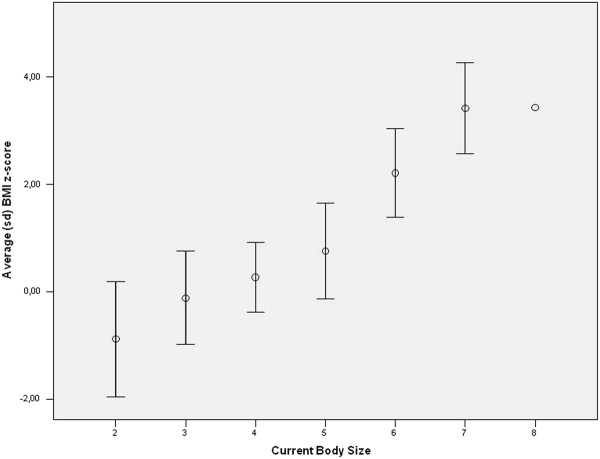
**Distribution of BMI z-score by Current Body Size for boys**. Florianopolis, Santa Catarina, Brazil, 2005.

**Figure 3:**
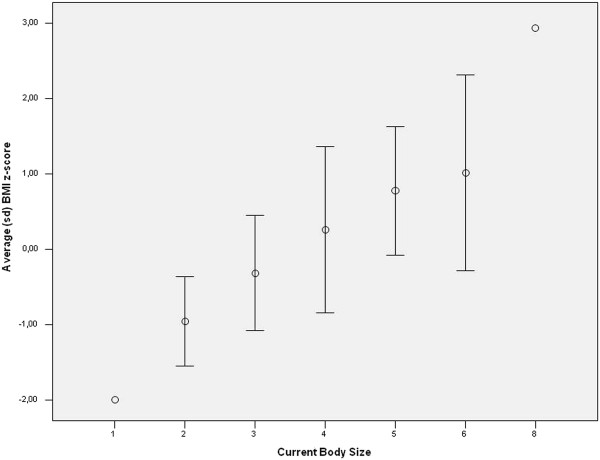
**Distribution of BMI z-score by Current Body Size for girls**. Florianopolis, Santa Catarina, Brazil, 2005.

Figures 2 and 3 show, for each sex, the means scores and standard deviation for BMI z-scores by Current Body Contour.

Correlation values were 0.62 (p < 0.001) for boys and 0.54 (p < 0.001) for girls. These correlation values indicate a positive linear trend between the variables BMI z-score and current body size, corroborating the hypothesis that the larger the body size score, the higher the BMI z-score.

**Figure 4:**
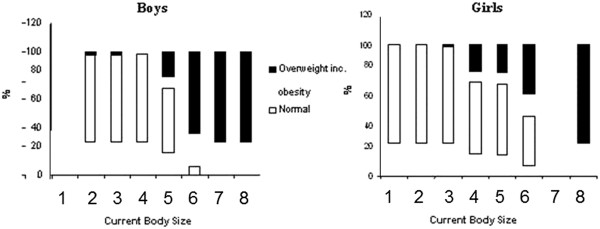
**Distribution of boys and girls according to nutritional status classification and Current Body Size**. Florianopolis, Santa Catarina, Brazil, 2005.

Figure 4 shows the distribution of children according to nutritional status classification and Current Body Size.

The coefficient of correlation between the module for the Degree of Dissatisfaction with Body Contour (DDBS) and BMI z-score was 0.37 (p < 0.001) for girls and -0.01 (p > 0.9) for boys. This shows that there is a trend to linearity between these variables in girls, whereas this trend was not identified in boys. In girls, therefore, the higher the BMI z-score the greater the body dissatisfaction.

## Discussion

The present study analyzed the construct validity for the figure rating scale put forward by Childress et al. [18] for Brazilian adolescents from ten to nineteen years old. In this study, the nutritional status was assessed only in terms of normal or overweight because there were no underweight adolescents. The data suggest construct validity of the scale because the following hypotheses are confirmed: 1) positive correlation between Current Body Size and BMI z-score in both sexes; 2) greater degree of body dissatisfaction (in module) by BMI z-score value in girls.

The last hypothesis was not expected to be proven in boys. Data from scientific research show that underweight or overweight boys are at greater risk of body dissatisfaction [11,12,15]. The hypothesis was expected to be proven for girls since the trend to the slim ideal begins in girlhood and accompanies women into adult life. Several studies show that the ideal of the female body image, both for adolescents and for adults, is predominantly geared toward weight loss, associated with the ideal female esthetic of slimness [27-29].

The use of figure rating scales is recurrent among researchers studying issues of body image and body dissatisfaction in populations of children and adolescents. Data from studies show that from the age of eight children have reliable responses in identifying body shape. However, there is still a lack of data about the validity of figure rating scales [28], since those that exist suggest correlations with variables such as social comparison, social and family support, the presence of eating disorders and BMI.

Wertheim and coworkers [30] found a correlation of 0.69 between the body size identified by female adolescents and BMI. The scale used in that study is different from that used in the present study. However, both are similar with regard to the larger number of figures (9 and 8 respectively) and subtle gradations from one figure to the next, which provides a greater range of choice. The similar correlations also suggest that the scales probably measure the same phenomenon. Other figure rating scales deemed valid in the scientific literature have an 0.41 [31] and 0.53 [32] correlation with BMI. In Wal & Thelen's study [32] the correlation of BMI with body dissatisfaction in adolescent females was 0.42, similar to the present study (0.37).

The figure rating scale used in the present study was constructed by Childress et al [18], adapted from the figure rating scales produced by Stunkard & Sorensen [19]. The adaptation aimed to enable the Stunkard scale to be used in evaluating body image and body dissatisfaction in children and adolescents. Interest in the Childress et al. scale [18] is due to the fact that the Stunkard and Sorensen figures [19] have been validated for the Brazilian adult population [2], and it was therefore important to validate an instrument with similar psychometric properties, at least regarding the construction of the instrument. In the validation study by Scagliusi et al. [2] the Spearman correlation between Current Body Size and BMI was 0.76, which indicates that the scale used for the Brazilian adult population and the scale adapted for use in Brazilian adolescents are possibly psychometrically equivalent.

The scientific community has been interested in the discussion on body image and body dimensions, above all body dissatisfaction, since the mid-twentieth century. Special importance has been given in this context to the issue of changes in body image over the several stages of a person's life, from childhood, through puberty and adolescence, to adult life. Aspects such as predictors, the consequences of body dissatisfaction, and an understanding of the processes that lead to changes in body image-related phenomena therefore lie within the scope of researchers interested in the topic [33].

The present study is therefore important in the discussion of changes in body image and body dissatisfaction over several life cycles in the Brazilian population, since it enables researchers to use an instrument for Brazilian adolescents that is psychometrically similar to the instrument that has already been validated for the adult population. The results of this study suggest that information obtained by the use of the Childress et al. scale [18] to evaluate body image in adolescents has considerable validity, and thus enables inferences to be drawn about the latent construct in question.

Although we described the reasons for choosing the scale adapted by adapted by Childress et al [18], it is also important to mention the existence of other relevant available tools well recognized in the literature such as the Veron-Guidry and Williamson's tool [34,35]. These findings indicate construct validity for the Childress figure rating scale [18] when used in 10-19-year-old Brazilian adolescents.

The present study thus describes the first evidence of a figure rating scale for Brazilian adolescents. We may consider our findings as a tool able to perform an association between body image dissatisfaction and BMI by means of novel statistical approaches such as spline function [36]. Further studies should be carried out to verify the validity of criterion and reliability of the Childress et al. figure rating scale [18] for Brazilian adolescents.

## Conclusion

The present study showed that both male and female individuals with greater BMI z-scores choose larger current body sizes. Furthermore, it was shown that girls with larger BMI z-scores show higher body dissatisfaction scores. This correlation is not valid for boys.

## Competing interests

The authors declare that they have no competing interests.

## Authors' contributions

All authors participated in the acquisition of data and revision of the manuscript. All authors determined the design, performed the statistical analysis, interpreted the data and drafted the manuscript. All authors read and gave final approval for the version submitted for publication.
